# Estimating safe maximum levels of vitamins and minerals in fortified foods and food supplements

**DOI:** 10.1007/s00394-016-1288-8

**Published:** 2016-08-08

**Authors:** Albert Flynn, Laura Kehoe, Áine Hennessy, Janette Walton

**Affiliations:** School of Food and Nutritional Sciences, University College Cork, Cork, Ireland

**Keywords:** Vitamins, Minerals, Safe maximum levels, Fortified foods, Supplements, Irish national nutrition surveys

## Abstract

**Purpose:**

To show how safe maximum levels (SML) of vitamins and minerals in fortified foods and supplements may be estimated in population subgroups.

**Methods:**

SML were estimated for adults and 7- to 10-year-old children for six nutrients (retinol, vitamins B6, D and E, folic acid, iron and calcium) using data on usual daily nutrient intakes from Irish national nutrition surveys.

**Results:**

SML of nutrients in supplements were lower for children than for adults, except for calcium and iron. Daily energy intake from fortified foods in high consumers (95th percentile) varied by nutrient from 138 to 342 kcal in adults and 40–309 kcal in children. SML (/100 kcal) of nutrients in fortified food were lower for children than adults for vitamins B6 and D, higher for vitamin E, with little difference for other nutrients. Including 25 % ‘overage’ for nutrients in fortified foods and supplements had little effect on SML. Nutritionally significant amounts of these nutrients can be added safely to supplements and fortified foods for these population subgroups. The estimated SML of nutrients in fortified foods and supplements may be considered safe for these population subgroups over the long term given the food composition and dietary patterns prevailing in the respective dietary surveys.

**Conclusions:**

This risk assessment approach shows how nutrient intake data may be used to estimate, for population subgroups, the SML for vitamins and minerals in both fortified foods and supplements, separately, each taking into account the intake from other dietary sources.

## Introduction

European Union (EU) legislation has provided for the harmonisation of regulations on the vitamin and mineral content of food supplements and fortified foods, including, among other aspects, the setting of (safe) maximum amounts of vitamins and minerals in these products, and lays down the criteria for their setting [1–4]. However, (safe) maximum amounts have not been set at EU level or in Ireland to date.

The legislation specifies that (safe) maximum amounts of vitamins and minerals in supplements and fortified foods will be set taking into account the upper safe levels [or tolerable upper intake levels (ULs)] established by scientific risk assessment and population reference intakes, as well as intakes of vitamins and minerals from other dietary sources. UL has been defined as ‘the maximum level of total chronic daily intake of a nutrient (from all sources) judged to be unlikely to pose a risk of adverse health effects to humans’ [5]. Because the same food supplements and fortified foods may be consumed by different population subgroups, the setting of (safe) maximum amounts must take into account what is safe for the whole population of consumers. This requires the estimation, where possible, of safe maximum levels (SML) for different population subgroups, particularly those who may be more sensitive (e.g. young children) [3, 4]. Estimates of SML are specific to prevailing food composition and dietary patterns in a population subgroup which may change over time, and policy makers need to anticipate such potential changes when setting (safe) maximum amounts that will be protective over future years.

A number of possible approaches have been proposed for the estimation of SML in fortified foods [6–11] and in supplements [7, 8, 11–13]. These are based on estimates of the maximum amounts of nutrients in foods and supplements that do not give rise to total intakes that exceed the UL, even in high consumers. All proposed approaches rely on estimates of intakes of nutrients from conventional (non-fortified) foods, fortified foods and food supplements derived from population dietary surveys. In some cases, these estimates include a provision for potential future changes in nutrient intakes, e.g. from increased consumption of fortified foods in countries where fortification practice has been limited in the past. For estimation of SML in fortified foods, the lack of data on the separate contribution of these dietary sources to intakes of vitamins and minerals and, in particular, on the contribution of fortified foods to intakes of energy and nutrients, has been a significant limitation of some of these approaches. This has led to the use of generally conservative assumptions and additional safety factors to derive estimates of SML in fortified foods that minimise the risk of excess intakes in population subgroups but may underestimate the SML for these nutrients. On the other hand, assumptions have been shown to be too liberal in some cases, e.g. estimates of the energy consumed from foods fortified with folic acid in young children in the Netherlands [14].

The Irish national nutrition surveys (www.iuna.net) have collected detailed data on food consumption and food composition, including supplements and fortified foods at brand level, for children and adults. These surveys show that fortified foods are widely consumed by children and adults [15–18] while supplements (mainly multivitamins/minerals) are used regularly by about 28 % of adults aged 18–64 years [19] and about 25 % children aged 5–12 years [20]. The availability of these data facilitates the simultaneous estimation of SML of vitamins and minerals in fortified foods and supplements. A preliminary outline of the approach used here was presented at a workshop in Gubbio, Italy, in 2008 [21].

The aim of this study was to show how the SML of vitamins and minerals in fortified foods and supplements may be estimated in population subgroups. In addition, the study explores the factors that may influence the estimates of the respective SML as well as the data needed for their estimation.

## Methods

### Selection of nutrients and dietary reference values

The vitamins and minerals selected for this analysis are added to both fortified foods and food supplements and have established UL, i.e. retinol, vitamins D, E and B6, (synthetic) folic acid, calcium and iron. UL for retinol, vitamins D, E and B6, folic acid and calcium (adults) was taken from the EU Scientific Committee on Food and the European Food Safety Authority [5, 22, 23], and for iron and calcium (children) from the US Food and Nutrition Board, Institute of Medicine [24, 25]. For retinol, UL for men was applied to usual intake data; however, for women of childbearing age UL is based on acute intakes (for teratogenicity) [5] and this was applied to daily intake in this age group estimated for each observation day. The UL for (synthetic) folic acid for children was used in this analysis for information only as published values [5] are derived from adult UL based on an adverse effect in older adults that is not considered to occur in children [26, 27], i.e. masking of megaloblastic anaemia in (undiagnosed) vitamin B12 deficiency and exacerbation of vitamin B12 deficiency-related neurological complications and cognitive decline. Nutrient reference values (NRV) for food labelling for vitamins and minerals were taken from the European Commission [28] and for energy from EFSA [29].

### Dietary data

This analysis was based on dietary intake data from two nationally representative dietary surveys in Ireland—the National Adult Nutrition Survey 2008–2010 (NANS) [30] and the National Children’s Food Survey 2003–2004 (NCFS) [31]. Adults aged 18–64 years (*n* = 1274) and children aged 7–10 years (*n* = 298) were selected for the study to represent population subgroups with different nutrient intakes from fortified foods and supplements and different UL for most of the selected nutrients.

The NANS and the NCFS established databases of food and drink consumption in representative samples of Irish adults aged 18–90 years and children aged 5–12 years, respectively. Study samples were representative of the respective population subgroups with respect to age, sex, social class and geographical location. A 4-day semi-weighed food record (including at least one weekend day) was used to collect dietary intake data from 1500 adults (740 men, 760 women), and a 7-day weighed food record was used to collect dietary intake data from 594 children (293 boys, 301 girls). Response rates were 60 and 66 % for NANS and NCFS, respectively. For both surveys, intake data for foods, drinks and supplements were recorded at brand level. Supplement use was also assessed using a questionnaire (supplement use over the recording week, over the previous 4 weeks and over the previous year). Nutrient intakes from supplements were estimated from the food diary as there were only small differences between the proportion of the subjects who reported consuming supplements in the food diary (25 % children, 28 % adults) and the proportion who reported consuming supplements over the previous year (29 % children, 30 % adults). Additionally, both surveys involved a high level of researcher–participant interaction which included face–face visits (up to four per participant) by trained nutritionists. More detail is available for both surveys at www.iuna.net.

Both studies were conducted according to the guidelines laid down in the Declaration of Helsinki. Ethical approval was obtained for both studies from the Clinical Research Ethics Committee of the Cork Teaching Hospitals, University College Cork. Written informed consent was obtained from study participants (adults) and from parents/guardians (children).

### Food composition data

Energy and nutrient intakes from both surveys were estimated using the Weighed Intake Software Program V3.0 (WISP©, Tinuviel Software, Anglesey, UK), which contains data from UK food composition tables, McCance and Widdowson’s The Composition of Foods 6th Edition [32] and the Irish Food Composition Database [33]. The Irish Food Composition Database has been consistently updated during each Irish national dietary survey to reflect the most recent composition data for fortified foods, nutritional supplements, composite dishes and Irish brands consumed which were not adequately characterised by UK food composition tables. The accuracy of food composition, as well as consumption, was aided in both surveys by asking participants to retain food packaging during the survey period. Thus, for fortified foods and supplements, composition data were obtained from the product label.

### Identification of fortified foods

Fortified foods were identified by the presence of vitamins and/or minerals in the ingredient list on the food label. ‘Fortification’ refers to the voluntary addition of vitamins and minerals by food manufacturers and excludes (semi-) mandatory addition of vitamins A and D to fat spreads and skimmed milk to ensure ‘nutritional equivalence’ and addition of iron, calcium, thiamin and niacin to flour for the purposes of ‘restoration’. These additions are considered ‘indigenous’ for the purpose of this paper. Fat spreads and skimmed milk which were fortified with nutrients other than vitamins A and D were included as fortified; however, the vitamin A and D contents of these foods were not included as fortified sources of vitamins A and D.

### Estimation of SML

SML of nutrients in food supplements and in fortified foods were estimated for 18- to 64-year-old adults in the NANS and 7- to 10-year-old children in the NCFS using data on food composition and dietary intakes prevailing during the respective national surveys. SML may be considered as the maximum level of a nutrient in supplements or fortified foods judged to be unlikely to pose a risk of adverse health effects in a population subgroup when consumed according to prevailing food composition and dietary patterns. The approach taken in estimating SML in supplements and fortified foods is to ensure that the UL is not exceeded by consumers with high intakes (95th percentile) of the nutrient from other dietary sources. Use of the 95th percentile (rather than higher percentiles) is considered appropriate considering the conservatism implicit in the setting of UL (5) such that 5 % of the population exceeding the UL by a modest amount would be unlikely to give rise to a risk of adverse health effects in the population.

For food supplements, SML_S_ of nutrients were estimated from the difference between the UL and the 95th percentile of the usual daily intake of the nutrients from conventional (non-fortified) foods (CI) and fortified foods (FFI): (CI + FFI)_95_. This is expressed per daily amount of a food supplement.

$${\text{SML}}_{\text{S}}\, \left( {\text{per daily amount}} \right) = {\text{UL}} - \left( {\text{CI + FFI}} \right)_{ 9 5}$$

Conventional (non-fortified) foods include foods with (semi-) mandatory additions of vitamins and minerals (see above), but exclude supplements.

For fortified foods, SML_F_ of nutrients were estimated from the difference between the UL and the 95th percentile of the usual daily intake of the nutrients from conventional (non-fortified) foods (CI) and supplements (SI): (CI + SI)_95_.

$${\text{SML}}_{\text{F}} \, \left( {\text{per daily amount}} \right) = {\text{UL}} - \left( {\text{CI + SI}} \right)_{ 9 5}$$

This was also expressed per 100 kcal of usual daily energy intake from foods fortified with the specific nutrient for high consumers (95th percentile) of energy from those foods (EFF_95_).

$${\text{SML}}_{\text{F}} \, \left( {\text{per 100 kcal}} \right) = \left[ {{\text{UL }} - \left( {\text{CI + SI}} \right)_{ 9 5} } \right] /\left[ {{\text{EFF}}_{ 9 5} / 1 0 0} \right]$$

or

$$= {\text{SML}}_{\text{F}} \, \left( {\text{per daily amount}} \right) /\left( {{\text{EFF}}_{ 9 5} / 1 0 0} \right)$$

For retinol, SML_S_ and SML_F_ were based on usual nutrient intakes for men but for women of childbearing age SML were based on acute daily intakes of nutrients to reflect the different basis of the UL for this group.

### Data analyses

Analysis of the data was conducted using SAS Enterprise Guide© for Windows™ Version 6.1 (SAS Institute Inc., Cary, NC, USA) or SPSS© for Windows version 21.0 (SPSS Inc.). Usual intakes of vitamins and minerals and usual intakes of energy from foods fortified with each nutrient were estimated using the validated National Cancer Institute (NCI) method [34]. The NCI method was implemented in SAS macros (v2.1), which were downloaded from the website http://appliedresearch.cancer.gov/diet/usualintakes/macros.html (date of download: 27th July 2015). For nutrient intakes, the approach used was to first sum the observed micronutrient intakes from food and supplements per participant per observation day and subsequently adjust for within-person variation using the NCI method, a first add then shrink approach [35]. While this approach may have limitations if the distribution of intakes from supplements and foods combined is multi-modal which can result in nonsensical outcomes [36], multi-modal distributions were not apparent in nutrient intake data from NANS or NCFS. Furthermore, there was no evidence of nonsensical outcomes, e.g. all estimates of usual nutrient intake from food plus supplements were similar to or higher than from food sources only, while the 95th percentile estimates of usual intake with the NCI method were all similar to or lower than the 95th percentile estimates of intake obtained without applying the NCI method. Although the NCI method is based on the assumption of at least two independent (non-consecutive) days of dietary intake for at least a subsample of subjects, use of multiple (four to seven) consecutive days for each subject gives reliable values for P_95_ intakes. For example, for 7- to 10-year-old children P_95_ of usual total daily intakes of nutrients estimated using seven consecutive days for each subject was 98 % (range 95–101 %) of P_95_ for two non-consecutive days (observation days 1 and 7). For retinol in women of childbearing age (18–50 years), intakes were estimated for each observation day using SPSS.

The 95th percentile of usual daily intake (P_95_) of vitamins and minerals in adults (18–64 years) and children (7–10 years) was estimated separately for total intake, intake excluding supplements (CI + FFI) and intake excluding fortified foods (CI + SI). The 95th percentile of usual daily intakes of energy from fortified foods (EFF_95_) were estimated for each nutrient in kcal/d and as per cent of total energy intake (%TE) (calculated per individual per day before applying the NCI method) in adults (18–64 years) and children (7–10 years). For retinol in women of childbearing age, the 95th percentile of intakes of energy from fortified foods was based on intakes for each day using SPSS. Correlations were examined between mean daily intake of nutrients and per cent energy from foods fortified with the specific nutrient using SPSS to obtain Spearman r values.

As it is usual practice for manufacturers to include additional nutrients (‘overage’) in fortified foods and supplements to allow for losses during processing and shelf life [37–40], estimates of intakes of vitamins and minerals were also made assuming the nutrient content in fortified foods and supplements exceeded the declared value on the label by an average of 25 % at time of consumption, in order to explore the effects of ‘overage’ on SML. European Commission guidance [41] to EU Member States on the setting of tolerances for nutrient values declared on a label states that the accepted tolerances for vitamins or minerals added to foods and food supplements should be no more than 45 % (minerals) or 50 % (vitamins) greater than the labelled value and no more than 35 % (foods) or 20 % (food supplements) below the labelled value. The measured value should be within the tolerances around the declared value during the entire shelf life of the product.

Preliminary analyses excluding potential energy under-reporters had little effect on SML for food supplements or foods in adults or children, and it was decided therefore not to exclude under-reporters from further analyses. Under-reporters of energy intake were identified as having a ratio of energy intake to basal metabolic rate <1.1 for adults [42] and using separate cut-offs for children [43]. The potential effect of energy over-reporting was not assessed since estimates of percentage of over-reporters and underestimation of energy intake are reported to be low with dietary assessment by food record methods [44].

## Results

The 95th percentile of usual total daily intakes for adults and children were less than the UL for all nutrients investigated (Table 1), even when overage of 25 % for fortified foods and supplements was included, ranging from 9 to 14 % of the UL for vitamins D and E in adults and children, up to 65–66 % of the UL for iron and calcium in adults and 55–60 % UL for calcium and retinol in children. Except for calcium and iron, the margins between the 95th percentile usual daily intakes and UL were narrower for children than adults, reflecting the lower UL for children. For retinol in women of childbearing age, the 95th percentile of daily intake was 38 % of the UL. Significant positive correlations were observed between mean daily intake of nutrients and per cent energy from foods fortified with the specific nutrient. These were generally low for nutrients that may be obtained from conventional foods, fortified foods and supplements (Spearman r 0.24–0.38), but were higher for folic acid (Spearman r 0.68) which is obtained only from fortified foods and supplements.

**Table 1:** The 95th percentile of usual daily intakes of nutrients from total diet^a^, intakes excluding supplements^b^ and intakes excluding fortified foods^c^ in 18- to 64-year-old adults in NANS and 7- to 10-year-old children in NCFS

Nutrient	Adults (*n* = 1274)				Children (*n* = 298)			
	UL	(CI + FFI + SI)_95_	(CI + FFI)_95_	(CI + SI)_95_	UL	(CI + FFI + SI)_95_	(CI + FFI)_95_	(CI + SI)_95_
Retinol (µg)	3000	1197^d^ [1313]	847^d^ [877]	1108^d^ [1195]	1500	799 [895]	503 [520]	741 [820]
Retinol (µg)	3000	1128^e^ [1323]	761^e^ [775]	1100^e^ [1287]				
Vitamin D (µg)	100	11.2 [12.6]	7.6 [8.0]	10.0 [10.9]	50	6.1 [7.0]	3.7 [3.9]	5.4 [6.0]
Vitamin E (mg)	300	28.2 [31.3]	16.7 [17.5]	25.3 [27.4]	160	12.7 [13.8]	9.8 [10.0]	11.9 [12.7]
Vitamin B6 (mg)	25	7.8 [8.8]	4.6 [5.0]	6.4 [6.9]	10	3.1 [3.5]	2.9 [3.2]	2.2 [2.2]
Folic acid (µg)	1000	370 [462]	241 [302]	366 [457]	(400)	(181) [226]	(156) [195]	(23) [29]
Calcium (mg)	2500	1560 [1621]	1495 [1531]	1471 [1497]	2500	1342 [1380]	1325 [1358]	1250 [1255]
Iron (mg)	45	27.0 [29.8]	20.1 [21.5]	22.5 [23.8]	40	14.8 [16.6]	13.8 [15.5]	9.5 [9.8]

Tolerable upper intake levels (ULs) are provided for reference

Values in brackets [] include 25 % overage from fortified foods and supplements

^a^CI + FFI + SI: intakes from total diet, including conventional (non-fortified) foods (CI), fortified foods (FFI) and supplements (SI)

^b^CI + FFI: intakes from total diet, including fortified foods but excluding supplements

^c^CI + SI: intakes from total diet, including supplements but excluding fortified foods

^d^Retinol for men (*n* = 634)

^e^Retinol for women of childbearing age (*n* = 485)

Daily energy intake from fortified foods in high consumers (95th percentile or EFF_95_) varied by nutrient (Table 2). For example, for adults the 95th percentile of usual daily energy intake from foods fortified with retinol and folic acid was 138 and 342 kcal, respectively, corresponding to a range of 6.4–17.1 %TE or 6.9–17.1 %NRV (2000 kcal). In women of childbearing age, the 95th percentile of daily energy intake from foods fortified with retinol was 75 kcal (4.3 %TE or 3.8 %NRV). For children, the 95th percentile of usual daily energy intake from foods fortified with retinol and folic acid was 40 and 309 kcal, respectively, corresponding to a range of 2.5–17.9 %TE or 2.0–15.5 %NRV.

**Table 2:** The 95th percentile of usual daily intake of energy from fortified foods (EFF_95_) for nutrients in 18- to 64-year-old adults in NANS and 7- to 10-year-old children in NCFS

Nutrient	P_95_ of daily energy intake from fortified foods			
	Adults (*n* = 1274)		Children (*n* = 298)	
	kcal/d	% TE^a^	kcal/d	% TE^a^
Retinol	138^b^	6.4^b^	40	2.5
Retinol	75^c^	4.3^c^		
Vitamin D	160	8.8	132	8.3
Vitamin E	133	6.8	60	3.4
Vitamin B6	244	12.4	291	16.5
Folic acid	342	17.1	(309)	(17.9)
Calcium	140	7.3	163	9.8
Iron	243	12.1	280	16.0

^a^%TE = % of total daily energy intake

^b^Retinol for men (*n* = 634)

^c^Retinol for women of childbearing age (*n* = 485)

For supplements, estimates of SML per daily amount were lower for children than for adults for all nutrients, except calcium and iron for which the UL is similar for both age groups (Table 3). SML per daily amount were many multiples of the NRV for nutrients with a large margin between the UL and the NRV (e.g. vitamins D, E and B6), but they were closer to the NRV for retinol, folic acid, calcium and iron. Including 25 % ‘overage’ in fortified foods and supplements had relatively little effect on SML per daily amount in supplements for any nutrient; the maximum difference was −8 % for folic acid (adults) and −6 % for iron (children). For retinol in women of childbearing age, SML per daily amount in supplements was more than twice the NRV.

**Table 3:** Safe maximum level in supplements (SML_S_) per daily amount for nutrients in 18- to 64-year-old adults in NANS and 7- to 10-year-old children in NCFS, and the effect of including ‘overage’ of 25 % for nutrients in fortified foods and supplements

Nutrient	NRV	SML_S_ per daily amount			
		Adults (*n* = 1274)		Children (*n* = 298)	
		Without overage	With overage	Without overage	With overage
Retinol (µg)	800	2153^a^	2123^a^	997	980
Retinol (µg)	800	2239^b^	2225^b^		
Vitamin D (µg)	5	92.4	92.0	46.3	46.1
Vitamin E (mg)	12	283.3	282.5	150.2	150.0
Vitamin B6 (mg)	1.4	20.4	20.0	7.1	6.8
Folic acid (µg)	200	759	698	(244)	(205)
Calcium (mg)	800	1005	969	1175	1142
Iron (mg)	14	24.9	23.5	26.2	24.5

Nutrient reference values (NRV) are included for reference

^a^Retinol for men (*n* = 634)

^b^Retinol for women of childbearing age (*n* = 485)

For fortified foods, estimates of SML per daily amount followed a similar pattern to that for supplements (Table 4). SML per daily amount were lower for children than for adults for all nutrients, except calcium and iron, and were many multiples of the NRV for nutrients vitamins D, E, and B6 but were closer to the NRV for retinol, folic acid, calcium and iron. For retinol in women of childbearing age, SML per daily amount in fortified foods was more than twice the NRV. Including 25 % ‘overage’ in fortified foods and supplements had relatively little effect on SML per daily amount of fortified foods for any nutrient; the maximum difference was −14 % for folic acid (adults) and −10 % for retinol (children).

**Table 4:** Safe maximum levels in fortified foods (SML_F_) per daily amount for nutrients in 18- to 64-year-old adults in NANS and 7- to 10-year-old children in NCFS, and the effect of including ‘overage’ of 25 % for nutrients in fortified foods and supplements

Nutrient	NRV	SML_F_ per daily amount			
		Adults (*n* = 1274)		Children (*n* = 298)	
		Without overage	With overage	Without overage	With overage
Retinol (µg)	800	1892^a^	1805^a^	759	680
Retinol (µg)	800	1900^b^	1713^b^		
Vitamin D (µg)	5	90	89.1	44.6	44
Vitamin E (mg)	12	275	273	148	147
Vitamin B6 (mg)	1.4	18.6	18.1	7.8	7.8
Folic acid (µg)	200	634	543	(377)	(371)
Calcium (mg)	800	1029	1003	1250	1245
Iron (mg)	14	22.5	21.2	30.5	30.2

Nutrient reference values (NRV) are included for reference

^a^Retinol for men (*n* = 634)

^b^Retinol for women of childbearing age (*n* = 485)

For fortified foods, SML per 100 kcal were lower for children than for adults for vitamins B6 and D, higher for vitamin E, and there was little difference for retinol, calcium or iron (Table 5). Including 25 % ‘overage’ in fortified foods and supplements had relatively little effect on SML per 100 kcal of fortified foods for any nutrient; the maximum difference for adults was −14 % for folic acid and for children −10 % for retinol. In women of childbearing age, SML for retinol per 100 kcal of fortified food was more than three times the NRV and were little effected by including 25 % ‘overage’ in fortified foods and supplements.

**Table 5:** Safe maximum levels in fortified foods (SML_F_) per 100 kcal for nutrients in 18- to 64-year-old adults in NANS and 7- to 10-year-old children in NCFS, and the effect of including ‘overage’ of 25 % for nutrients in fortified foods and supplements

Nutrient	NRV	SML_F_ per 100 kcal			
		Adults (*n* = 1274)		Children (*n* = 298)	
		Without overage	With overage	Without overage	With overage
Retinol (µg)	800	1371^a^	1308^a^	1898	1700
Retinol (µg)	800	2533^b^	2284^b^		
Vitamin D (µg)	5	56.0	55.7	33.8	33.3
Vitamin E (mg)	12	207	205	247	246
Vitamin B6 (mg)	1.4	7.6	7.4	2.7	2.7
Folic acid (µg)	200	185	159	(122)	(120)
Calcium (mg)	800	735	716	767	764
Iron (mg)	14	9.3	8.7	10.9	10.8

Nutrient reference values (NRV) are included for reference

^a^Retinol for men (*n* = 634)

^b^Retinol for women of childbearing age (*n* = 485)

In order to explore the limitations if data are not available separately for nutrient intakes from fortified foods, SML in fortified foods were estimated using the 95th percentile of total daily intake of nutrients (including fortified foods) instead of the 95th percentile of total daily intake excluding fortified foods. SML per daily amount in fortified foods were reduced for all nutrients in adults and children. For adults the difference was −9 % or less for all nutrients except iron (−20 %), while for children the difference was −8 % or less for all nutrients except for iron (−17 %) and vitamin B6 (−12 %). Larger differences were observed for nutrients for which fortified foods contribute significantly to the 95th percentile of total intake.

## Discussion

This study shows how nutrient intake data may be used to estimate the SML for both fortified foods and supplements, separately, each taking into account the intake from other dietary sources. There is an inverse relationship between SML of a nutrient and intake from other dietary sources in high consumers, e.g. SML per daily amount of a nutrient in supplements decreases with increasing intake of the nutrient in high consumers from food sources (including fortified foods).

The SML of nutrients in fortified foods and supplements estimated by this method are levels that may be considered safe for the respective population subgroups over the long term, when the nutrients are consumed according to prevailing food composition and dietary patterns. The method of estimating SML is such that:

- high consumers of a nutrient from food sources (including fortified foods) will not exceed the UL if supplements contain ≤SML per daily amount for that nutrient and
- high consumers of a nutrient from conventional (non-fortified) foods plus supplements, who are also high consumers of foods fortified with that nutrient, will not exceed the UL if fortified foods contain ≤SML per 100 kcal for that nutrient.

The findings of this study should be considered in the context of the composition and patterns of consumption of supplements and fortified foods in Ireland prevailing at the time of the respective dietary surveys. Supplements (mainly multivitamins/minerals typically containing one NRV per daily amount) were used regularly by about 28 % of adults aged 18–64 years [19] and about 25 % of children aged 5–12 years [20]. Fortified foods were consumed by over 80 % of adults and almost all children, contributing 8–9 %TE and significant proportions of intakes of some micronutrients (20–30 % of iron and B vitamins and 15 % vitamin D in children, 15–25 % of iron and B vitamins and 13 % vitamin D in adults) [15–18]. Of the fortified foods consumed, ready-to-eat breakfast cereals (RTEBC) and related products predominated, contributing over half of the energy derived from fortified foods in adults and children, with lesser contributions for other categories such as beverages, breads, spreads, and milk and yogurt. Almost all RTEBC are fortified with iron and a range of B vitamins, with about half fortified with vitamin D. For added vitamins and minerals, typical levels in fortified foods are 20–40 % NRV per average serving. Folic acid was the nutrient most commonly added to fortified foods, while retinol was added to relatively few foods. There has been an increase in the consumption of fortified foods over recent years associated with increased numbers of fortified foods within a wider range of food categories, rather than to increased levels of addition of nutrients, and this has resulted in greater contributions from fortified foods to daily intakes of iron, folate and other B vitamins. However, there is evidence that the contribution of fortified foods to folic acid intake has decreased recently, associated with discontinuation of fortification of some fat spreads and breads [45].

The 95th percentile of the usual daily intakes of the selected vitamins and minerals in adults aged 18–64 years and children aged 7–10 years were well below the respective UL even allowing for overage of 25 % in fortified foods and supplements. This indicates that, with prevailing dietary patterns in these population subgroups, and at prevailing levels of these nutrients in fortified foods and supplements, there is little risk of adverse health effects associated with excess intakes. It should be noted that this assessment does not take into account possible future changes in composition of foods and supplements and in dietary patterns which policy makers need to consider when setting (safe) maximum amounts in fortified foods and supplements.

Estimates of SML per daily amount in supplements were lower for children than for adults for those nutrients with lower UL for children but not for calcium and iron, where the UL is similar for both age groups. For some nutrients (e.g. vitamins D, E and B6), the SML in supplements were many multiples of the NRV, but these values were closer to the NRV for retinol, folic acid, calcium and iron where the margins between the UL and the NRV are relatively small. Including 25 % ‘overage’ in fortified foods and supplements had relatively little effect on SML in supplements for adults or children, and SML exceeded the NRV for all nutrients. This indicates that, with prevailing food composition and dietary patterns, nutritionally significant amounts of these nutrients may be added safely to supplements for these age groups in Ireland, in agreement with other estimates [12, 13].

SML per 100 kcal of fortified food were lower for children than for adults for some nutrients (vitamins B6 and D), but higher for vitamin E, with little difference for retinol, calcium or iron. Including 25 % ‘overage’ in fortified foods and supplements had relatively little effect on SML per 100 kcal of fortified food which was not less than 60 % of the NRV for any nutrient for adults and children and exceeded the NRV by a considerable margin for some. Thus, with prevailing food composition and dietary patterns, nutritionally significant amounts of these nutrients can be added safely to foods and beverages for these population subgroups in Ireland. For example, with 25 % NRV per portion, a typical amount in fortified products [17], the proportion of NRV per 100 kcal would be 23 % for a breakfast cereal (110 kcal in a 30 g portion), 25 % for a yoghurt (100 kcal in 125 g portion) or 28 % for a beverage (90 kcal in 200 ml portion). These SML also exceed the minimum required for labelling as ‘source’ of nutrient, i.e. 15 % NRV per 100 g of food, 7.5 % NRV per 100 ml of beverage [28].

Daily energy intake from fortified foods in high consumers (95th percentile) is an important variable in the estimation of the SML per 100 kcal of fortified food. There are few data available for energy intake from fortified foods [6, 14, 17, 18] and approaches to the estimation of the SML in fortified foods to date have generally assumed the same value for all nutrients, for example, based on an assumed fraction of food that is potentially fortifiable [6, 9, 10]. This has been done when data on fortified food consumption were not available from dietary surveys and also to anticipate possible future increased consumption of fortified foods in countries where fortification practice has been limited in the past. However, this study showed that daily energy intake from fortified foods in high consumers varied considerably between nutrients, ranging in adults from 138 kcal for retinol to 342 kcal for folic acid and in children from 40 kcal for retinol to 309 kcal for folic acid. Furthermore, values were higher for children than for adults for some nutrients (vitamin B6, calcium and iron) and lower for others (retinol, vitamins D and E). Thus, it is not appropriate to assume a single value for energy intake from fortified foods for all nutrients when estimating SML per 100 kcal. These variations in energy intake from fortified foods reflect differences in patterns of intake of foods fortified with different nutrients [15–18] prevailing during the respective dietary surveys as described earlier. Since these may change over time with changing practices of food fortification [18], policy makers need to consider possible future changes when setting (safe) maximum amounts in fortified foods.

Estimation of SML in fortified foods by the approach proposed here may be limited by the availability of data on energy and nutrient intakes from fortified foods, as is often the case [46, 47]. While it is possible to obtain reasonable approximations of SML per daily amount (although conservative for some nutrients) using total daily intakes of nutrients with fortified foods included instead of excluded, estimation of SML per 100 kcal requires an estimate of daily energy intakes from fortified foods in high consumers for each nutrient. The estimates of daily energy intakes from fortified foods for adults and children in Ireland, a country with a long-standing, liberal policy on food fortification, may be a useful starting point for such estimations in other EU countries. Since fortification of foods often differs between product brands, it has been recommended that national dietary surveys should include data on both the consumption and composition of foods at brand level in order to facilitate adequate monitoring of fortified foods [47].

A strength of this study is its use of detailed data on consumption and composition of foods, including fortified foods, and supplements in nationally representative surveys. This facilitates estimation of intakes of energy and nutrients from fortified foods separately from non-fortified foods and supplements. This study also used a statistical method for estimating the distribution of usual intakes of nutrients and energy [34] which corrects the within-person variation arising from short-term dietary measurements over a limited number of days. This provides a more robust estimate of 95th percentile intakes of energy and nutrients and helps to avoid underestimating the SML. Limitations of the study include reliance on nutrient composition obtained from food labels for fortified foods and supplements and possible under-reporting of food intake which occurs in dietary surveys. However, these were addressed in the analyses by estimating SML including overage of 25 % in fortified foods and supplements and by demonstrating that analysis excluding energy under-reporters had little effect on the estimates of SML.

The SML for population subgroups may be used in setting (safe) maximum amounts of nutrients in fortified foods and supplements in the whole population of consumers. For this, there is a need to take account of younger children who have lower UL for some nutrients [3–6, 9, 10, 12, 13], as well as potential changes in the SML with changing food composition and patterns of dietary intake over time. Regular national dietary surveys are needed to monitor changes in the composition and consumption of fortified foods and supplements in order to assess the safety of micronutrient intakes in the population. For nutrients with no reported adverse effects at high intakes and with no UL (e.g. thiamin, riboflavin), there is no scientific basis for establishing SML and there may be no need for (safe) maximum amounts to be set for fortified foods or supplements for such nutrients [4]. This also applies to folic acid in children where UL are derived from adult UL based on an adverse effect in older adults that is not considered to occur in children [26, 27], i.e. masking of megaloblastic anaemia in (undiagnosed) vitamin B12 deficiency and exacerbation of vitamin B12 deficiency-related neurological complications and cognitive decline [5]. Although it has been proposed that folic acid may have other adverse effects, e.g. arising from the presence of unmetabolised folic acid in blood, no such effects have been confirmed [27, 48] and no UL has been established based on such possible adverse effects. For nutrients with established adverse effects but with insufficient data to establish UL, other approaches are needed for setting (safe) maximum amounts, e.g. a case-by-case qualitative risk management approach [13].

## Conclusions

This risk assessment approach shows how nutrient intake data may be used to estimate, for population subgroups, the SML for vitamins and minerals in both fortified foods and supplements, separately, each taking into account the intake from other dietary sources. The SML of nutrients in fortified foods and supplements estimated by this method are levels that may be considered safe for the respective population subgroups over the long term, when the nutrients are consumed according to prevailing food composition and dietary patterns. Such SML, together with appropriate allowances for possible future changes in food composition and dietary patterns, may be used by policy makers for setting maximum amounts of vitamins and minerals in fortified foods and supplements that are safe for the whole population of consumers. The study also emphasises the importance of collecting adequate data on composition and consumption of fortified foods and supplements in national dietary surveys on a regular basis in order to monitor the safety of micronutrient intakes.
